# Sentiment of Emojis

**DOI:** 10.1371/journal.pone.0144296

**Published:** 2015-12-07

**Authors:** Petra Kralj Novak, Jasmina Smailović, Borut Sluban, Igor Mozetič

**Affiliations:** Jožef Stefan Institute, Jamova 39, 1000 Ljubljana, Slovenia; University of Maribor, SLOVENIA

## Abstract

There is a new generation of emoticons, called emojis, that is increasingly being used in mobile communications and social media. In the past two years, over ten billion emojis were used on Twitter. Emojis are Unicode graphic symbols, used as a shorthand to express concepts and ideas. In contrast to the small number of well-known emoticons that carry clear emotional contents, there are hundreds of emojis. But what are their emotional contents? We provide the first emoji sentiment lexicon, called the Emoji Sentiment Ranking, and draw a sentiment map of the 751 most frequently used emojis. The sentiment of the emojis is computed from the sentiment of the tweets in which they occur. We engaged 83 human annotators to label over 1.6 million tweets in 13 European languages by the sentiment polarity (negative, neutral, or positive). About 4% of the annotated tweets contain emojis. The sentiment analysis of the emojis allows us to draw several interesting conclusions. It turns out that most of the emojis are positive, especially the most popular ones. The sentiment distribution of the tweets with and without emojis is significantly different. The inter-annotator agreement on the tweets with emojis is higher. Emojis tend to occur at the end of the tweets, and their sentiment polarity increases with the distance. We observe no significant differences in the emoji rankings between the 13 languages and the Emoji Sentiment Ranking. Consequently, we propose our Emoji Sentiment Ranking as a European language-independent resource for automated sentiment analysis. Finally, the paper provides a formalization of sentiment and a novel visualization in the form of a sentiment bar.

## Introduction

An **emoticon**, such as ;-), is shorthand for a facial expression. It allows the author to express her/his feelings, moods and emotions, and augments a written message with non-verbal elements. It helps to draw the reader’s attention, and enhances and improves the understanding of the message. An **emoji** is a step further, developed with modern communication technologies that facilitate more expressive messages. An emoji is a graphic symbol, ideogram, that represents not only facial expressions, but also concepts and ideas, such as celebration, weather, vehicles and buildings, food and drink, animals and plants, or emotions, feelings, and activities.

Emojis on smartphones, in chat, and email applications have become extremely popular worldwide. For example, Instagram, an online mobile photo-sharing, video-sharing and social-networking platform, reported in March 2015 that nearly half of the texts on Instagram contained emojis [1]. The use of emojis on the SwiftKey Android and iOS keybords, for devices such as smartphones and tablets, was analyzed in the SwiftKey Emoji Report [2], where a great variety in the popularity of individual emojis, and even between countries, was reported. However, to the best of our knowledge, no large-scale analysis of the emotional content of emojis has been conducted so far.

Sentiment analysis is the field of study that analyzes people’s opinions, sentiments, evaluations, attitudes, and emotions from a text [3, 4]. In analyzing short informal texts, such as tweets, blogs or comments, it turns out that the emoticons provide a crucial piece of information [5–12]. However, emojis have not been exploited so far, and no resource with emoji sentiment information has been provided.

In this paper we present the Emoji Sentiment Ranking, the first emoji sentiment lexicon of 751 emojis. The lexicon was constructed from over 1.6 million tweets in 13 European languages, annotated for sentiment by human annotators. In the corpus, probably the largest set of manually annotated tweets, 4% of the tweets contained emojis. The sentiment of the emojis was computed from the sentiment of the tweets in which they occur, and reflects the actual use of emojis in a context.

**Background**. An emoticon is a short sequence of characters, typically punctuation symbols. The use of emoticons can be traced back to the 19^*th*^ century, when they were used in casual and humorous writing. The first use of emoticons in the digital era is attributed to professor Scott Fahlman, in a message on the computer-science message board of Carnegie Mellon University, on September 19, 1982. In his message, Fahlman proposed to use :-) and :-( to distinguish jokes from more serious posts. Within a few months, the use of emoticons had spread, and the set of emoticons was extended with hugs and kisses, by using characters found on a typical keyboard. A decade later, emoticons had found their way into everyday digital communications and have now become a paralanguage of the web [6].

The word ‘emoji’ literally means ‘picture character’ in Japanese. Emojis emerged in Japan at the end of the 20^*th*^ century to facilitate digital communication. A number of Japanese carriers (Softbank, KDDI, DoCoMo) provided their own implementations, with incompatible encoding schemes. Emojis were first standardized in Unicode 6.0 [13]—the core emoji set consisted of 722 characters. However, Apple’s support for emojis on the iPhone, in 2010, led to global popularity. An additional set of about 250 emojis was included in Unicode 7.0 [14] in 2014. As of August 2015, Unicode 8.0 [15] defines a list of 1281 single- or double-character emoji symbols.

**Related work**. Sentiment analysis, or opinion mining, is the computational study of people’s opinions, sentiments, emotions, and attitudes. It is one of the most active research areas in natural-language processing and is also extensively studied in data mining, web mining, and text mining [3, 4]. The growing importance of sentiment analysis coincides with the growth of social media, such as Twitter, Facebook, book reviews, forum discussions, blogs, etc.

The basis of many sentiment-analysis approaches is the sentiment lexicons, with the words and phrases classified as conveying positive or negative sentiments. Several general-purpose lexicons of subjectivity and sentiment have been constructed. Most sentiment-analysis research focuses on English text and, consequently, most of the resources developed (such as sentiment lexicons and corpora) are in English. One such lexical resource, explicitly devised to support sentiment classification and opinion mining, is SentiWordNet 3.0 [16]. SentiWordNet extends the well-known WordNet [17] by associating each synset with three numerical scores, describing how ‘objective’, ‘positive’, and ‘negative’ the terms in the synset are.

Emoticons have proved crucial in the automated sentiment classification of informal texts [5–12]. In an early work [10], a basic distinction between positive and negative emoticons was used to automatically generate positive and negative samples of texts. These samples were then used to train and test sentiment-classification models using machine learning techniques. The early results suggested that the sentiment conveyed by emoticons is both domain and topic independent. In later work, these findings were applied to automatically construct sets of positive and negative tweets [8, 18, 19], and sets of tweets with alternative sentiment categories, such as the angry and sad emotional states [11]. Such emoticon-labeled sets are then used to automatically train the sentiment classifiers. Emoticons can also be exploited to extend the more common features used in text mining, such as sentiment-carrying words. A small set of emoticons has already been used as additional features for polarity classification [8, 20]. A sentiment-analysis framework that takes explicitly into account the information conveyed by emoticons is proposed in [6].

There is also research that analyzes graphical emoticons and their sentiment, or employs them in a sentiment classification task. The authors in [21] manually mapped the emoticons from Unicode 8.0 to nine emotional categories and performed the sentiment classification of tweets, using both emoticons and bag-of-words as features. Ganesan et al. [22] presents a system for adding the graphical emoticons to text as an illustration of the written emotions.

Several studies have analyzed emotional contagion through posts on Facebook and showed that the emotions in the posts of online friends influence the emotions expressed in newly generated content [23–26]. Gruzd et al. [27] examined the spreading of emotional content on Twitter and found that the positive posts are retweeted more often than the negative ones. It would be interesting to examine how the presence of emojis in tweets affects the spread of emotions on Twitter, i.e., to relate our study to the field of emotional contagion [28].

**Contributions**. Emojis, a new generation of emoticons, are increasingly being used in social media. Tweets, blogs and comments are analyzed to estimate the emotional attitude of a large fraction of the population to various issues. An emoji sentiment lexicon, provided as a result of this study, is a valuable resource for automated sentiment analysis. The Emoji Sentiment Ranking has a format similar to SentiWordNet [16], a publicly available resource for opinion mining, used in more than 700 applications and studies so far, according to Google Scholar. In addition to a public resource, the paper provides an in-depth analysis of several aspects of emoji sentiment. We draw a sentiment map of the 751 emojis, compare the differences between the tweets with and without emojis, the differences between the more and less frequent emojis, their positions in tweets, and the differences between their use in the 13 languages. Finally, a formalization of sentiment and a novel visualization in the form of a sentiment bar are presented.

## Results and Discussion

### Emoji sentiment lexicon

The sentiment of emojis is computed from the sentiment of tweets. A large pool of tweets, in 13 European languages, was labeled for sentiment by 83 native speakers. Sentiment labels can take one of three ordered values: *negative* ≺ *neutral* ≺ *positive*. A sentiment label, *c*, is formally a discrete, 3-valued variable *c* ∈ {−1, 0, +1}. An emoji is assigned a sentiment from all the tweets in which it occurs. First, for each emoji, we form a discrete probability distribution (*p*_−_, *p*_0_, *p*_+_). The sentiment score $\bar{s}$ of the emoji is then computed as the mean of the distribution. The components of the distribution, i.e., *p*_−_, *p*_0_, and *p*_+_ denote the negativity, neutrality, and positivity of the emoji, respectively. The probability *p*_*c*_ is estimated from the number of occurrences, *N*, of the emoji in tweets with the label *c*. Note that an emoji can occur multiple times in a single tweet, and we count all the occurrences. A more detailed formalization of the sentiment representation can be found in the Methods section.

**Fig 1:**
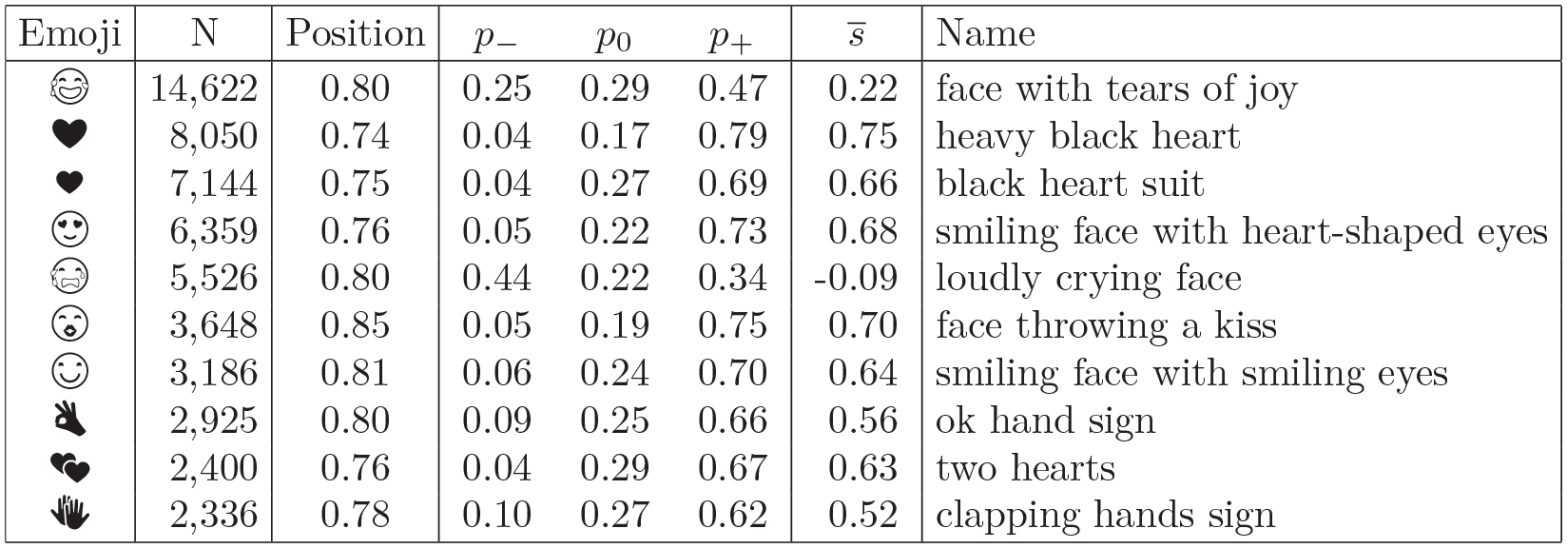
Top 10 emojis. Emojis are ordered by the number of occurrences *N*. The average *position* ranges from 0 (the beginning of the tweets) to 1 (the end of the tweets). *p*_*c*_, *c* ∈ {−1, 0, +1}, are the negativity, neutrality, and positivity, respectively. $\bar{s}$ is the sentiment score.

We thus form a sentiment lexicon of the 751 most frequent emojis, called the Emoji Sentiment Ranking. The complete Emoji Sentiment Ranking is available as a web page at http://kt.ijs.si/data/Emoji_sentiment_ranking/. The 10 most frequently used emojis from the lexicon are shown in Fig 1.

First we address the question of whether the emojis in our lexicon are representative. We checked **Emojitracker** (http://emojitracker.com/), a website that monitors the use of emojis on Twitter in realtime. In the past two years, Emojitracker has detected almost 10 billion emojis on Twitter! From the ratio of the number of emoji occurrences and tweets in our dataset (∼2.3), we estimate that there were about 4 billion tweets with emojis. In our dataset of about 70,000 tweets, we found 969 different emojis, 721 of them in common with Emojitracker.

We compared the emojis in both sets, ordered by the number of occurrences, using Pearson’s [29] and Spearman’s rank [30] correlation. We successively shorten our list of emojis by cutting off the least-frequent emojis. The results for two thresholds, *N* ≥ 1 and 5, with the highest correlation coefficients, are shown in Table 1. Both correlation coefficients are high, significant at the 1% level, thus confirming that our list of emojis is indeed representative of their general use on Twitter. Between the two options, we decided to select the list of emojis with at least 5 occurrences, resulting in the lexicon of 751 emojis. The sentiment scores for the emojis with fewer then 5 occurrences are not very reliable.

**Table 1:** Overlap with Emojitracker. Correlations are between the occurrences of emojis in the Emoji Sentiment Ranking and Emojitracker, for two minimum occurrence thresholds. The numbers in parenthesis are the emojis that are common in both sets. The correlation values, significant at the 1% level, are indicated by *.

	Tweets with emojis	Different emojis used	Pearson correlation	Spearman rank correlation
Emojitracker	∼4 billion	845	/	/
Emoji Sent. Rank.				
*N* ≥ 1	69,673	969 (721)	0.945*	0.897*
Emoji Sent. Rank.				
*N* ≥ 5	69,546	**751** (608)	0.944*	0.898*

### Emoji sentiment map

**Fig 2:**
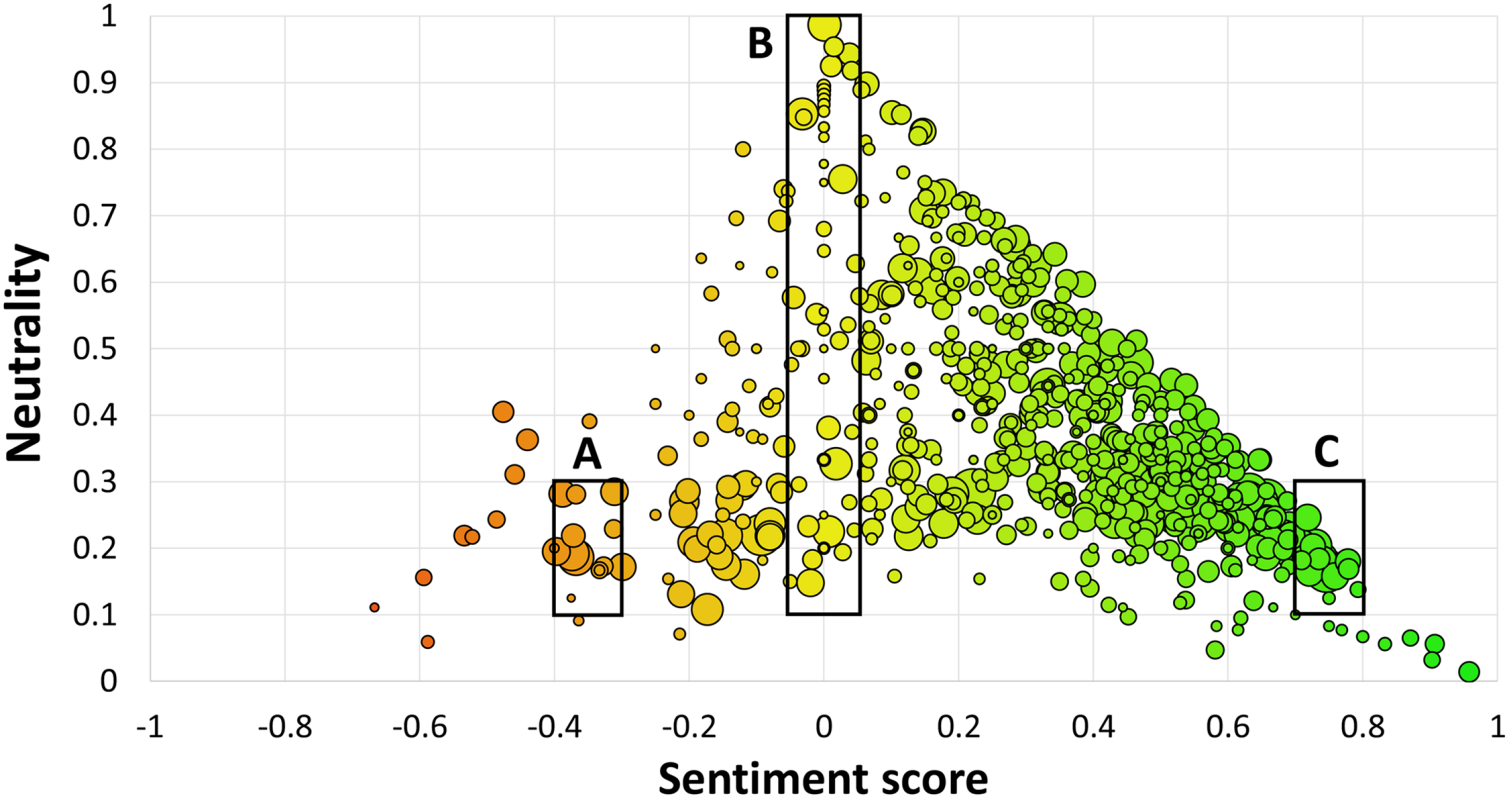
Sentiment map of the 751 emojis. Left: negative (red), right: positive (green), top: neutral (yellow). Bubble size is proportional to log_10_ of the emoji occurrences in the Emoji Sentiment Ranking. Sections A, B, and C are references to the zoomed-in panels in Fig 3.

Before we analyze the properties of the tweets with emojis, we first discuss two visualizations of the Emoji Sentiment Ranking. Fig 2 shows the overall map of the 751 emojis. The position of an emoji is determined by its sentiment score $\bar{s}$ and its neutrality *p*_0_. The sentiment score $\bar{s}$ is in the range (−1, +1) and is computed as *p*_+_ − *p*_−_. The more positive emojis are on the right-hand side of the map (green), while the negative ones are on the left-hand side (red). The neutral emojis (yellow) span a whole band around $\bar{s}=0$. The emojis are prevailingly positive, the mean sentiment score is +0.3 (see the Sentiment distribution subsection). The bubble sizes are proportional to the number of occurrences.

**Fig 3:**
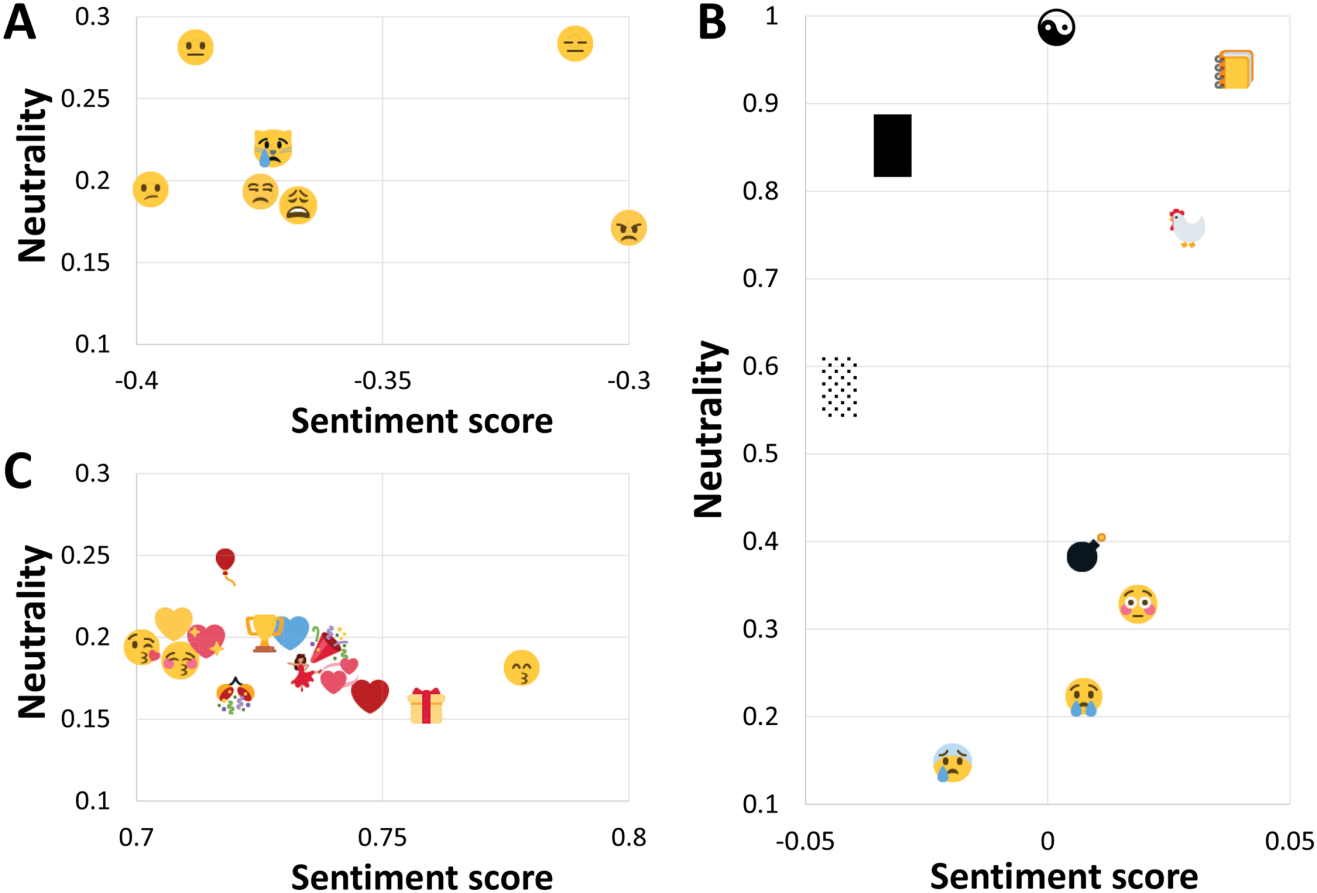
Emojis in sections A, B, and C of Fig 2. Shown are emojis that occur at least 100 times in the Emoji Sentiment Ranking. Panel **A**: negative emojis, panel **B**: neutral (top) and bipolar (bottom) emojis, panel **C**: positive emojis.

A more detailed view of some actual emojis on the map is shown in Fig 3. The most frequent negative emojis (panel A) are sad faces. On the other hand, the most frequent positive emojis (panel C) are not only happy faces, but also hearts, party symbols, a wrapped present, and a trophy. Even more interesting are the neutral emojis (panel B). All of them have a sentiment score around 0, but the neutrality *p*_0_ ranges between 0 and 1. The bottom two, with low *p*_0_ (face with cold sweat, crying face), are bipolar, with a high negativity and positivity, where *p*_−_ ≈ *p*_+_. The middle two (flushed face, bomb) have a uniform sentiment distribution, where *p*_−_ ≈ *p*_0_ ≈ *p*_+_. The top ones, with high *p*_0_, are neutral indeed, symbolized by the yin yang symbol at the very top.

### Tweets with and without emojis

In this subsection we analyze the interplay of the human perception of tweets that are with and without emojis. If we consider the sentiment of a tweet as a rough approximation of its emotional content, we can ask two questions. Are the tweets with emojis more emotionally loaded? Does the presence of emojis in tweets have an impact on the human emotional perception of the tweets? We do not draw any causal conclusions, but report the results of two experiments that indicate that the answer to both questions is positive.

First, we compare all the manually labeled tweets that are with and without emojis. From the distribution of the negative, neutral, and positive tweets in both sets, we compute the mean, standard deviation (sd), and standard error of the mean (sem). The results are shown in Table 2.

**Table 2:** Sentiment of tweets with and without emojis. For each set, the mean, sd and sem are computed from the distribution of negative, neutral, and positive tweets.

Sentiment	Tweets with emojis	Tweets without emojis
*Negative*	12,156 (17,5%)	410,301 (26,1%)
*Neutral*	19,938 (28,6%)	587,337 (37,3%)
*Positive*	37,579 (53,9%)	576,424 (36,6%)
Total	69,673	1,574,062
Mean	+0.365	+0.106
sd, sem	0.762, 0.0029	0.785, 0.0006

We test the null hypothesis that the two populations have equal means. We apply Welch’s t-test [31] for two samples with unequal variances and sizes. We are aware that the two populations might not be normally distributed, but Welch’s t-test is robust for skewed distributions, and even more so for large sample sizes [32]. With *t* = 87, the degrees of freedom ≫ 100 (due to large sample sizes), and the p-value ≈ 0, the null hypothesis can be rejected. We can conclude, with high confidence, that the tweets with and without emojis have significantly different sentiment means. Additionally, the tweets with emojis are significantly more positive (mean = +0.365) than the tweets without emojis (mean = +0.106).

Next, we compare the agreement of the human annotators on the tweets with and without emojis. The Twitter sentiment classification is not an easy task and humans often disagree on the sentiment labels of controversial tweets. During the process of annotating the 1.6 million tweets, we found that even individual annotators are not consistent with themselves. Therefore, we systematically distributed a fraction of the tweets to be annotated twice in order to estimate the level of agreement. This annotator self-agreement is a good indicator of the reliability of the annotator. The inter-annotator agreement, on the other hand, indicates the difficulty of the task. In the case of emojis, our goal is to verify whether their presence in tweets correlates with a higher inter-annotator agreement.

There are a number of measures to estimate the inter-annotator agreement. We apply three of them from two different fields, to ensure robust estimates. The first one, Krippendorff’s *Alpha*-reliability [33], generalizes several specialized agreement measures. When the annotators are in perfect agreement, *Alpha* = 1, and when the level of agreement equals the agreement by chance, *Alpha* = 0. We applied an instance of *Alpha* that takes into account the ordering of labels and assigns a higher penalty to more extreme disagreements. For example, a disagreement between the *negative* and the *positive* sentiment is four times as costly as that between the *neutral* and *positive*.

The simplest measure of agreement is the joint probability of agreement, also known as *Accuracy*, when evaluating classification models. *Accuracy* is the number of equally labeled tweets by different annotators, divided by the total number of tweets labeled twice. It assumes the data labels are unordered (nominal) and does not take into account the agreement by chance, but it is easy to interpret.

The third measure comes from the field of machine learning. It is used to evaluate the performance of classification models against a test set, where the true sentiment label is known. The measure, $\bar{F_{1}}$(−, +), is a standard measure of performance, specifically designed for a 3-valued sentiment classification [12], where the *negative* (−) and *positive* (+) sentiments are considered more important than the *neutral* one. Here, we adapt it to estimate the agreement of a pair of annotators.

Table 3 gives the results of the inter-annotator agreements on the tweets with and without emojis. Coincidence matrices for both cases are in the Methods section. All three measures of agreement, *Alpha*, *Accuracy*, and $\bar{F_{1}}$(−, +), are considerably higher for the tweets with emojis, by 21%, 10%, and 17%, respectively. We do not give any statistical-significance results, but it seems safe to conclude that the presence of emojis has a positive impact on the emotional perception of the tweets by humans. After all, this is probably the main reason why they are used in the first place.

**Table 3:** Inter-annotator agreement on tweets with and without emojis. The agreement is computed in terms of three measures over a subset of tweets that were labeled by two different annotators.

Agreement measure	Tweets with emojis	Tweets without emojis
*Alpha*	0.597	0.495
*Accuracy*	0.641	0.583
$\bar{F_{1}}$(−, +)	0.698	0.598
No. of tweets annotated twice	3,547	52,027

### Sentiment distribution

**Fig 4:**
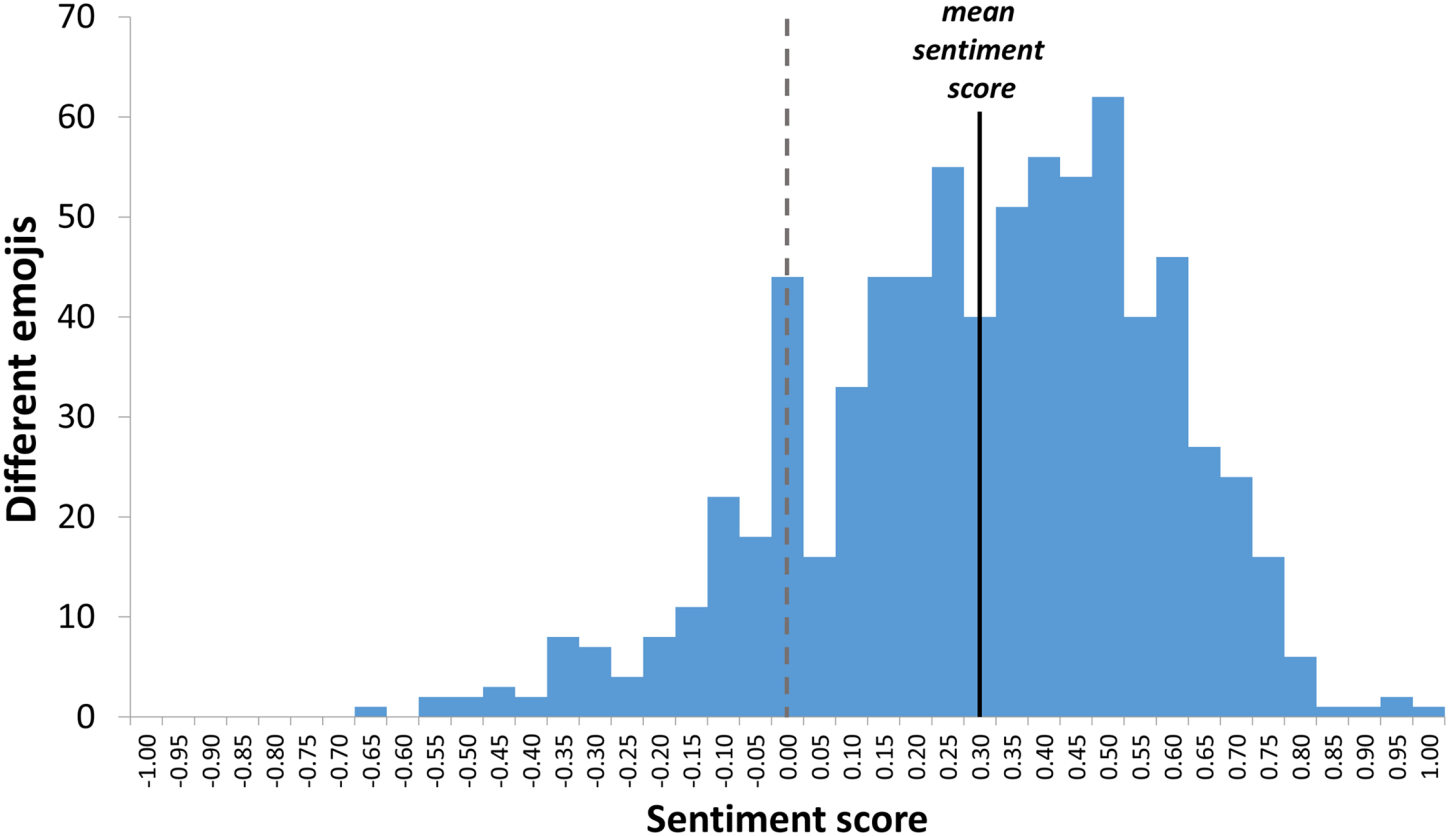
Distribution of emojis by sentiment score. The mean sentiment score of the 751 emojis (in bins of 0.05) is +0.305.

In this subsection we analyze the sentiment distribution of the emojis with respect to the frequency of their use. The question we address is the following: Are the more frequently used emojis more emotionally loaded? First, in Fig 4 we show the sentiment distribution of the 751 emojis, regardless of their frequencies. It is evident that the sentiment score of the emojis is approximately normally distributed, with mean = +0.3, prevailingly positive.

**Fig 5:**
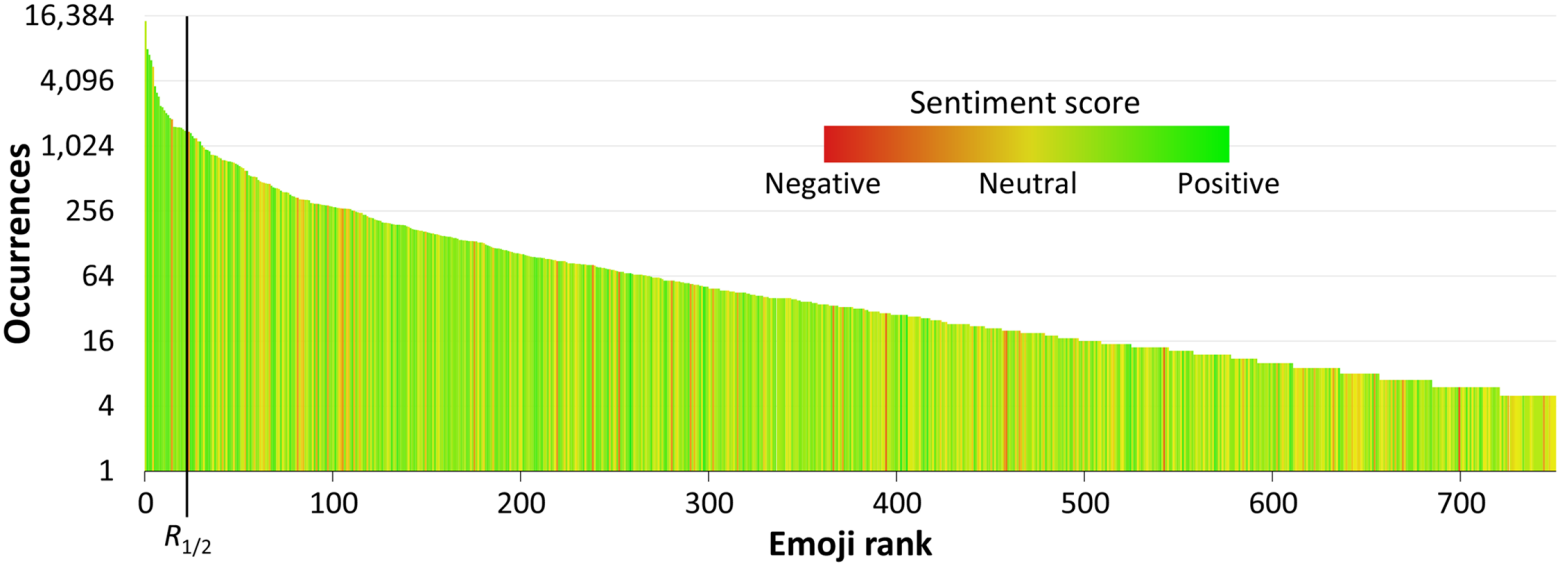
Distribution of occurrences and sentiment of the 751 emojis. The emojis are ranked by their occurrence (log scale). The column color indicates the sentiment score. The partitioning into two equally weighted halfs is indicated by a line at *R*_1/2_. The first 33 emojis are zoomed-in in Fig 6.

**Fig 6:**
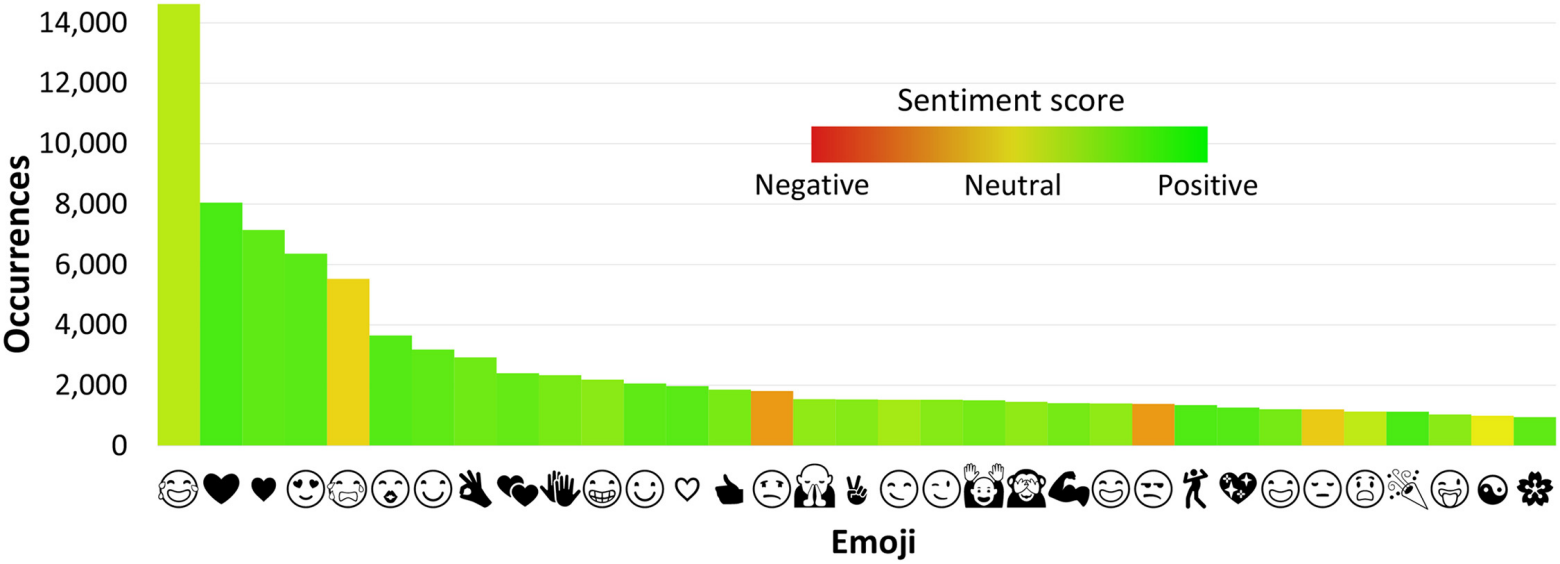
Top 33 emojis by occurrence. Column color represents the emoji sentiment score.

In Fig 5 we rank the emojis by the number of their occurrences in tweets. The sentiment score of each emoji is indicated by the color. The zoomed-in section of the first 33 emojis is in Fig 6.

We did not thoroughly analyze the frequency-rank distribution of the emojis. A quick analysis suggests that the data follows a power law with an exponential cutoff at a rank of about 200. Using a maximum-likelihood estimator [34], the exponent of the power law is estimated to be −1.3, a relatively extreme exponent. Even more relevant is the distribution of the emojis on Emojitracker, but this remains a subject of further research. Here we concentrate on the sentiment distribution.

We define a cumulative distribution function CDF(*R*) of rank *R* over a set of ranked emojis as:

$$\begin{array}{c} \text{CDF}\left(R\right)=N\left(r\leq R\right)=\sum_{r\leq R}N\left(r\right)\;, \end{array}$$

where *r* denotes the rank of an emoji, and *N*(*r*) the number of occurrences of the emoji at rank *r*. In order to compare the higher-ranked emojis (more frequent) with the lower-ranked ones (less frequent), we define a midpoint rank *R*_1/2_, such that:

$$\begin{array}{c} N\left(1\leq r\leq R_{1/2}\right)\approx N\left(R_{1/2}<r\leq 751\right)\;. \end{array}$$

The midpoint rank *R*_1/2_ partitions the emojis into two subsets with an approximately equal cumulative number of occurrences. In the case of the Emoji Sentiment Ranking, the midpoint is at *R*_1/2_ = 23.

We compute the mean sentiment, sd, and sem of the more frequent and the less frequent emojis. The results are shown in Table 4.

**Table 4:** Comparison of the more-frequent with the less-frequent emojis. The emojis (*r*) ranked by occurrence *N*(*r*) are partitioned into two halves with approximately the same cumulative number of occurrences.

	**1st half** (*r* ≤ 23)	**2nd half** (23 < *r*)	Total
Different emojis	23	728	751
Occurrences (∑*N*(*r*))	77,969	78,488	156,457
Sentiment mean	+0.463	+0.311	+0.387
sd, sem	0.280, 0.0010	0.319, 0.0011	0.300, 0.0008

We test the null hypothesis that the two populations of emojis have equal mean sentiment scores. Again, we apply Welch’s t-test for two samples with unequal variances, but similar sizes. With *t* = 100, the degrees of freedom ≫100 (due to large sample sizes), and the p-value ≈ 0, the null hypothesis can be rejected. We can conclude, with high confidence, that the more-frequent emojis are significantly more positive than the less-frequent ones.

This result supports the thesis that the emojis that are used more often are more emotionally loaded, but we cannot draw any causal conclusion. Are they more positive because they are more often used in positive tweets, or are they more frequently used, because they are more positive?

### Sentiment and emoji position

**Fig 7:**
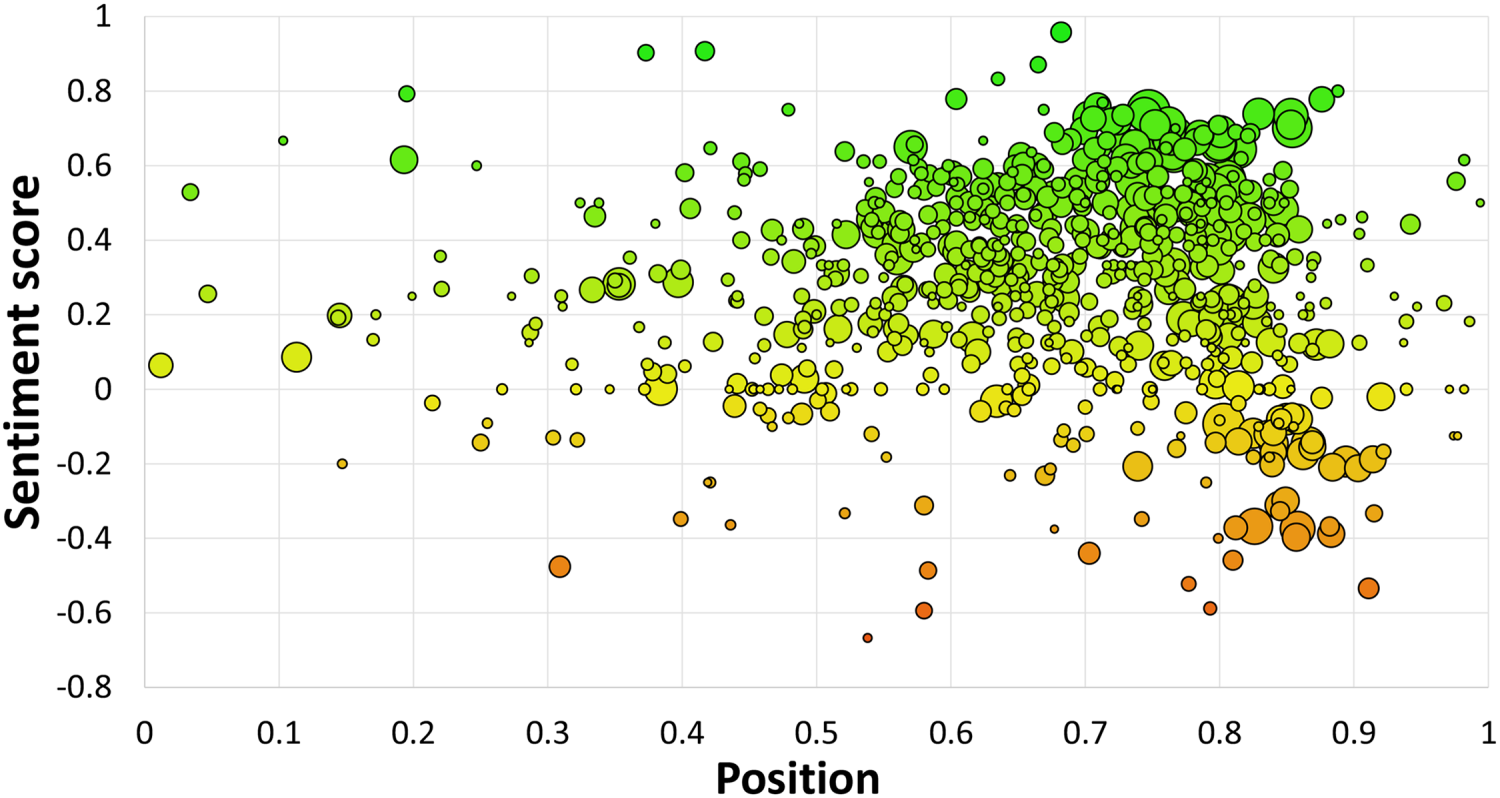
Average positions of the 751 emojis in tweets. Bubble size is proportional to log_10_ of the emoji occurrences in the Emoji Sentiment Ranking. Left: the beginning of tweets, right: the end of tweets, bottom: negative (red), top: positive (green).

Where are the emojis typically placed in tweets? Emoticons such as :-) are used sparsely and typically at the very end of a sentence. Emojis, on the other hand, appear in groups and not only at the end of the tweets. Fig 7 shows the average positions of the 751 emojis in the tweets. On average, an emoji is placed at 2/3 of the length of a tweet.

**Fig 8:**
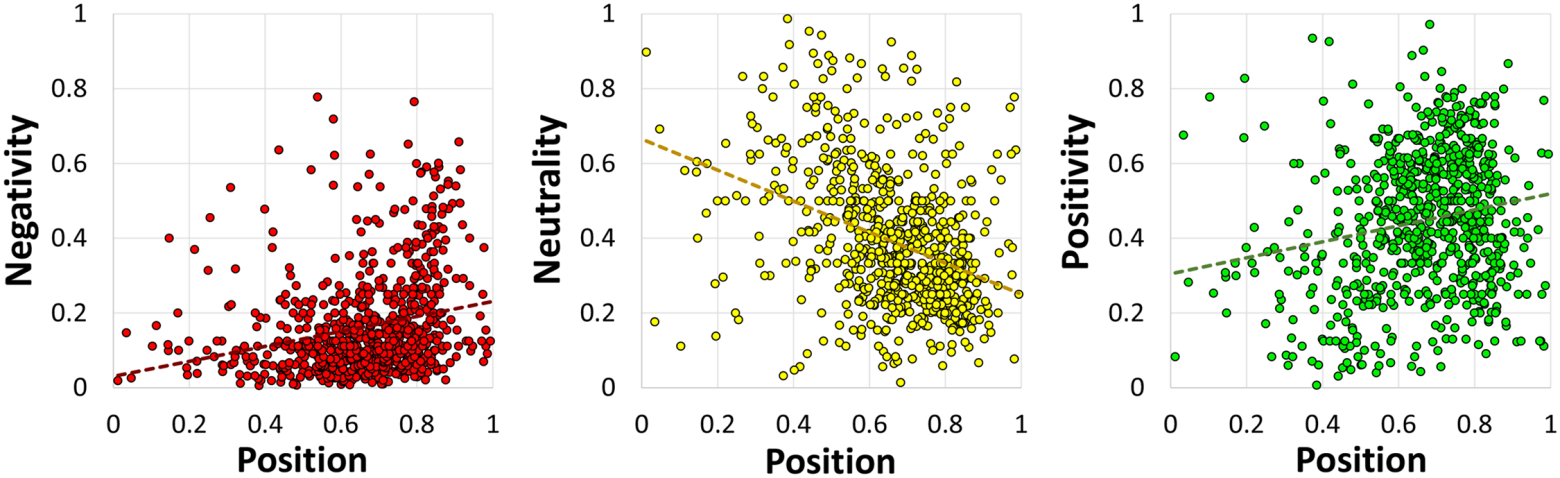
Negativity, neutrality, and positivity regressed with position (from left to right). The trendlines are functions *p*_*c*_(*d*) of the distance *d* from the beginning of the tweets.

Fig 7 also indicates the sentiment of an emoji in relation to its position. In Fig 8 we decompose the sentiment into its three constituent components and show the regression trendlines.

The linear regression functions in Fig 8 have the following forms:

$$\begin{array}{c} \text{negativity}:\;\;\;p_{-}\left(d\right)=0.20d+0.03\;\;\left(R^{2}=0.06\right)\;, \end{array}$$

$$\begin{array}{c} \text{neutrality}:\;\;\;p_{0}\left(d\right)=-0.41d+0.66\;\;\left(R^{2}=0.14\right)\;, \end{array}$$

$$\begin{array}{c} \text{positivity}:\;\;\;p_{+}\left(d\right)=0.21d+0.30\;\;\left(R^{2}=0.04\right)\;, \end{array}$$

where *d* is the distance from the beginning of the tweets. The functions do not fit the data very well, but they give some useful insight. At any distance *d*, and for any subset of emojis, the component probabilities add up to 1:

$$\begin{array}{c} \sum_{c}p_{c}\left(d\right)=1 \end{array}$$

However, the negativity and positivity increase with the distance, whereas the neutrality decreases. This means that more emotionally loaded emojis, either negative or positive, tend to occur towards the end of the tweets.

### Emojis in different languages

In the final subsection we analyze the use of emojis in the 13 languages processed in this study. Can the Emoji Sentiment Ranking be considered a universal resource, at least for European languages? Is the sentiment ranking between the different languages significantly different? The results in Table 5 indicate that the answer to the first question is positive and that there is no evidence of significant differences between the languages.

**Table 5:** Emoji sentiment in different languages. The languages are ordered by the number of different emojis used. Correlations are between the sentiment scores of emojis in the 13 languages and the Emoji Sentiment Ranking. The correlation values, significant at the 1% level, are indicated by *.

	Tweets with emojis	Different emojis used	Pearson correlation	Spearman rank correlation
Emoji Sent. Rank.	69,546	751	/	/
English	19,819	511	0.834*	0.819*
Spanish	22,063	448	0.552*	0.573*
Polish	8,112	253	0.810*	0.783*
Russian	5,007	221	0.777*	0.756*
Hungarian	2,324	176	0.588*	0.612*
German	3,062	142	0.782*	0.783*
Swedish	2,797	139	0.702*	0.674*
Ser/Cro/Bos	2,096	123	0.708*	0.615*
Slovak	1,526	108	0.620*	0.499*
Slovenian	996	66	0.526*	0.541*
Portuguese	796	56	0.410*	0.429*
Bulgarian	607	36	0.557*	0.443*
Albanian	341	19	0.363 *	0.416 *

For each language, we form a list of emojis used in the collected tweets of the language, cut off the emojis with fewer than 5 occurrences (the same threshold as applied to the overall Emoji Sentiment Ranking), and compute their sentiment score. We compute the correlation coefficients between the Emoji Sentiment Ranking and the individual languages. As can be seen in Table 5, the number of emojis actually used in the different languages (above the threshold) drops considerably. However, their sentiment scores and ranking remain stable. Both Pearson’s correlation and Spearman’s rank correlation are relatively high, and significant for all the languages, except Albanian. This result is biased towards languages with more tweets since they have a larger share in the joint Emoji Sentiment Ranking. An alternative test might compare individual languages and the Emoji Sentiment Ranking with the language removed. However, as a first approximation, it seems reasonable to use the Emoji Sentiment Ranking as a universal, language-independent resource, at least for European languages.

## Conclusions

In this paper we describe the construction of an emoji sentiment lexicon, the Emoji Sentiment Ranking, the first such publicly available resource. We have formalized and analyzed the sentiment properties of the emojis in depth and highlighted some interesting conclusions.

The data that enabled these analyses, 1.6 million annotated tweets in 13 different languages, is a valuable resource with many other useful applications. In particular, we are constructing sentiment-classification models for different languages, and applying them to various tasks. The Slovenian and Bulgarian language-sentiment models were already applied to monitor the mood on Twitter during political elections in realtime [35]. The English sentiment model was used to compare the sentiment leanings of different retweet network communities towards various environmental topics [36]. A domain-specific English sentiment model (from another set of financial tweets) was applied to analyze the effects of Twitter sentiment on stock prices [37]. Yet another English sentiment model was constructed by combining a large set of general, emoticon-labeled tweets with domain-specific financial tweets, and tested for Granger causality on the Baidu stocks [38]. The same methodology of manual text annotations, automated model construction, and sentiment classification was also applied to Facebook comments in Italian, where the emotional dynamics in the spreading of conspiracy theories was studied [26].

The sentiment annotation of tweets by humans is expensive. Emoticons were already used as a proxy for the sentiment labels of tweets. We expect that the Emoji Sentiment Ranking will turn out to be a valuable resource for helping humans during the annotation process, or even to automatically label the tweets with emojis for sentiment. In a lexicon-based approach to sentiment analysis, the emoji lexicon can be used in combination with a lexicon of sentiment-bearing words. Alternatively, an emoji with already-known sentiment can act as a seed to transfer the sentiment to the words in proximity. Such a corpus-based approach can be used for an automated corpus construction for feature generation [12], and then applied to train a sentiment classifier.

There are other dimensions of sentiment that are beyond the one-dimensional scale from negativity to positivity and worth exploring. The expressiveness of the emojis allows us to assign them more subtle emotional aspects, such as anger, happiness, or sadness, and some shallow semantics, such as activities, locations, or objects of interest. An additional structuring of the emojis can be derived from correlations between their sentiment, e.g., various versions of hearts expressing love. However, we consider the interplay between the emojis and the text to be one of the most promising directions for future work. Not only the position of an emoji, but certainly its textual context is also important in determining the role of the emoji as an amplifier and modifier of the meaning.

In the future, it will be interesting to monitor how the use of emojis is growing, and if textual communication is increasingly being replaced by a pictorial language. Also, the sentiment and meaning of emojis evolve over time. It might be interesting to investigate the convergence of agreement on the meaning of controversial emojis, and to study the underpinnings of the corresponding social processes.

## Methods

### Ethics statement

The tweets were collected through the public Twitter API and are subject to the Twitter terms and conditions. The sentiment annotations were supported by the Goldfinch platform, provided by Sowa Labs (http://www.sowalabs.com). The human annotators were engaged for the purpose, and were aware that their annotations will be used to construct the sentiment-classification models, and to estimate the inter-annotator agreement and the annotator self-agreement.

### Data collection

The main source of the data used in this study is a collection of tweets, in 13 European languages, collected between April 2013 and February 2015. Most of the tweets (except English) were collected during a joint project with Gama System (http://www.gama-system.si), using their PerceptionAnalytics platform (http://www.perceptionanalytics.net). The tweets of selected languages were collected through Twitter Search API, by specifying the geolocations of the largest cities. For English tweets, we used Twitter Streaming API (a random sample of 1% of all public tweets), and filtered out the English posts.

We have engaged 83 native speakers (except for English) to manually annotate for sentiment over 1.6 million of the collected tweets. The annotation process was supported by the Goldfinch platform designed specifically for sentiment annotation of short texts (such as Twitter posts, Facebook comments, …). The annotators were instructed to label each tweet as either *negative*, *neutral*, or *positive*, by estimating the emotional attitude of the user who posted the tweet. They could also skip the inappropriate or irrelevant tweets. The breakdown of the annotated tweets by language is in Table 6.

**Table 6:** Tweets annotated for sentiment in different languages. Languages are in alphabetical order, Ser/Cro/Bos denotes a union of tweets in Serbian, Croatian and Bosnian.

Language	No. of tweets	No. of annotators
Albanian	53,005	13
Bulgarian	67,169	18
English	103,034	9
German	109,130	5
Hungarian	68,505	1
Polish	223,574	8
Portuguese	157,393	1
Russian	107,773	1
Ser/Cro/Bos	215,657	13
Slovak	70,425	1
Slovenian	133,935	7
Spanish	275,588	5
Swedish	58,547	1
Total	1,643,735	83

Another source of data comes from Emojitracker (http://emojitracker.com/). Emojitracker monitors and counts the number of emojis used on Twitter in realtime. It has been active since July 2013, and so far it has detected over 10 billion emoji occurrences. We downloaded the current count of emoji occurrences as of June 2015. This data is used to estimate how representative is our sample of emojis in the annotated tweets.

The data from both sources is available in a public language-resource repository clarin.si at http://hdl.handle.net/11356/1048. There are two data tables, in an open csv format, one for the Emoji Sentiment Ranking, and the other from Emojitracker. The tables list all the emojis found, their occurrences, and, in the case of the Emoji Sentiment Ranking, also their numbers in the negative, neutral, and positive tweets. From this data, the Emoji Sentiment Ranking web page at http://kt.ijs.si/data/Emoji_sentiment_ranking/ is automatically generated.

### Emoji Unicode symbols

The exact definition of what constitutes an emoji symbol is still emerging. In particular, there is some discrepancy between our set of emojis and the emojis tracked by Emojitracker. Also, during the writing of this paper, in August 2015, the Unicode consortium published a new set of emojis, the **Unicode Emoji Charts** (http://www.unicode.org/emoji/).

The set of emojis in our Emoji Sentiment Ranking follows the Unicode standard version 8 [15] and consists of all the single-character symbols from the Unicode category ‘Symbol, Other’ (abbreviated **[So]**) that appear in our tweets. Emojitracker, on the other hand, also tracks some double-character symbols (10 Country Flags, and 11 Combining Enclosing Keycaps), but does not track all the **[So]** symbols that appear in our data. In particular, 49 Dingbats, 46 Miscellaneous Symbols, 38 Box Drawings, 28 Geometric Shapes, 21 Enclosed Alphanumerics, 20 Enclosed Alphanumeric Supplements, and 13 Arrows are not tracked. The Unicode Emoji Charts have introduced even more new emoji symbols, in particular an exhaustive list of 257 double-character Country Flags. A comparison of the overlaps and differences in the emoji symbol specifications between the three sources is in Tables 7 and 8.

**Table 7:** Types and numbers of emoji symbols. **[So]** is an abbreviation for the Unicode category ‘Symbol, Other’.

	No. of all emoji symbols	single character	[So]
			non-[So]
		double character	flags
			keycaps
**Emoji Sentiment Ranking**	969	969	969
			0
		0	0
			0
**Emojitracker**	845	824	812
			12
		21	10
			11
**Unicode Emoji Charts**	1281	1012	995
			17
		269	257
			12

**Table 8:** Overlaps and differences for emojis from the three data sources. A table entry is the number of emojis in (∈), or missing (∉) from a data source. *N*(*Single*, *Double*) denotes the total number *N* of emoji symbols, partitioned into the *Single*- and *Double*-character symbols, respectively.

		**Emoji Sentiment Ranking**		
		∈	∉	Total
**Emojitracker**	∈	721 (721, 0)	124 (103, 21)	845 (824, 21)
	∉	248 (248, 0)	/	/
**Unicode**	∈	734 (734, 0)	547 (278, 269)	1281 (1012, 269)
**Emoji Charts**	∉	235 (235, 0)	/	/
Total		969 (969, 0)	/	

The emoji symbols that are not common to all the three data sources are relatively infrequent. The highest-ranking emoji in Emojitracker, which is absent from our data, has the rank 157 (double exclamation mark). The highest-ranking emoji in the Emoji Sentiment Ranking, not tracked by Emojitracker, has the rank 13 (white heart suit). Additionally, we noticed that we missed three characters from the **[So]** category: ‘degree sign’, ‘numero sign’, and ‘trade mark sign’. However, only the ‘trade mark sign’ (with 257 occurrences in our data) is also considered by the Emojitracker and the Unicode Emoji Charts. Despite these minor differences in the emoji sets, all our results remain valid. However, in the next version of the Emoji Sentiment Ranking we plan to extend our set to double-character symbols, and consider all the emojis from the Unicode Emoji Charts as an authoritative source.

### Sentiment formalization

The sentiment of an individual tweet can be *negative*, *neutral*, or *positive*. Formally, we represent it by a discrete, 3-valued variable, *c*, which denotes the sentiment class:

$$\begin{array}{c} c\in \{-1,0,+1\} \end{array}$$

This variable models well our assumptions about the ordering of the sentiment values and the distances between them.

An object of Twitter posts to which we attribute sentiment (an emoji in our case, but it can also be a stock [37], a political party [35], a discussion topic [26, 36], etc.) occurs in several tweets. A discrete distribution:

$$\begin{array}{c} N\left(c\right)\;,\;\sum_{c}N\left(c\right)=N\;,\;c\in \left\{-1,0,+1\right\}\;, \end{array}$$

captures the sentiment distribution for the set of relevant tweets. *N* denotes the number of all the occurrences of the object in the tweets, and *N*(*c*) are the occurrences in tweets with the sentiment label *c*. From the above we form a discrete probability distribution:

$$\begin{array}{c} \left(p_{-},p_{0},p_{+}\right)\;,\;\sum_{c}p_{c}=1\;. \end{array}$$

For convenience, we use the following abbreviations:

$$\begin{array}{c} p_{-}=p\left(-1\right)\;,\;p_{0}=p\left(0\right)\;,\;p_{+}=p\left(+1\right)\;, \end{array}$$

where *p*_−_, *p*_0_, and *p*_+_ denote the **negativity**, **neutrality**, and **positivity** of the object (an emoji in our case), respectively. In SentiWordNet [16], the term **objectivity** is used instead of the neutrality *p*_0_. The **subjectivity** can then be defined as *p*_−_+*p*_+_ [39].

Typically, probabilities are estimated from relative frequencies, *p*_*c*_ = *N*(*c*)/*N*. For large samples, such estimates are good approximations. Often, however, and in particular in our case, we are dealing with small samples, e.g., *N* = 5. In such situations, it is better to use the *Laplace estimate* (also known as the *rule of succession*) to estimate the probability [40]:

$$\begin{array}{c} p_{c}=\frac{N(c)+1}{N+k}\;,\;\left(\text{for}\;\text{large}\;N:\;p_{c}\approx \frac{N(c)}{N}\right)\;. \end{array}$$

The constant *k* in the denominator is the cardinality of the class, in our case *k* = |*c*| = 3. The Laplace estimate assumes a prior uniform distribution, which makes sense when the sample size is small.

Once we have a discrete probability distribution, with properly estimated probabilities, we can compute its mean:

$$\begin{array}{c} \bar{x}=\sum_{c}p_{c}\cdot c\;. \end{array}$$

We define the **sentiment score**, $\bar{s}$, as the mean of the discrete probability distribution:

$$\begin{array}{c} \bar{s}=-1\cdot p_{-}+0\cdot p_{0}+1\cdot p_{+}=p_{+}-p_{-}\;. \end{array}$$

The sentiment score has the range: $-1<\bar{s}<+1$.

The standard deviation of a discrete probability distribution is:

$$\begin{array}{c} \text{SD}=\sqrt{\sum_{c}p_{c}\cdot {\left(c-\bar{x}\right)}^{2}}\;, \end{array}$$

and the standard error of the mean is:

$$\begin{array}{c} \text{SEM}=\frac{\text{SD}}{\sqrt{N}}\;. \end{array}$$

### Sentiment bar

**Fig 9:**
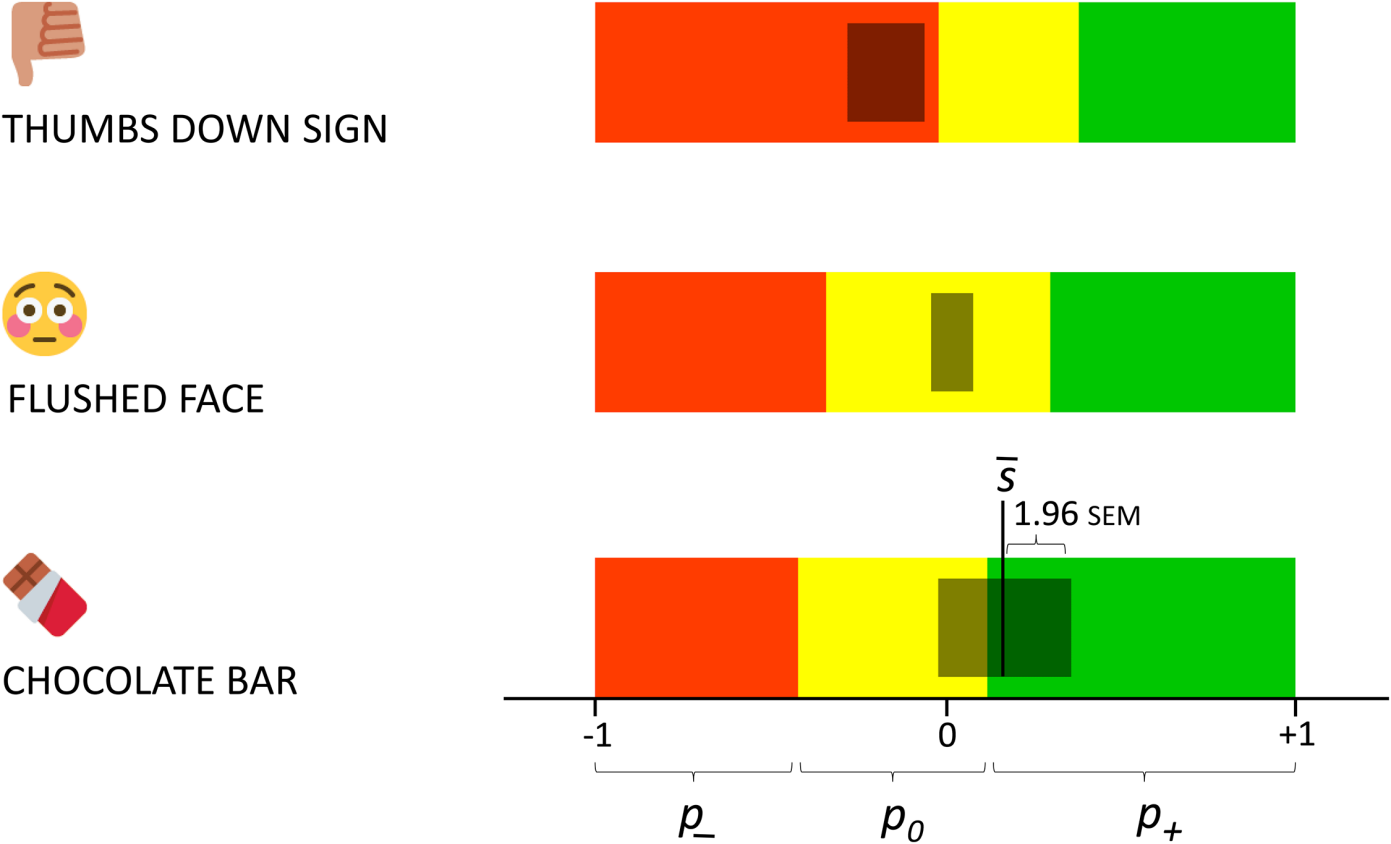
Sentiment bars of the ‘thumbs down sign’, ‘flushed face’, and ‘chocolate bar’ emojis. The colored bar extends from −1 to +1, the range of the sentiment score $\bar{s}$. The grey bar is centered at $\bar{s}$ and extended for ±1.96SEM, but never beyond the range of $\bar{s}$. Colored parts are proportional to negativity (*p*_−_, red), neutrality (*p*_0_, yellow), and positivity (*p*_+_, green).

The sentiment bar is a useful, novel visualization of the sentiment attributed to an emoji (see http://kt.ijs.si/data/Emoji_sentiment_ranking/ for examples). In a single image, it captures all the sentiment properties, computed from the sentiment distribution of the emoji occurrences: $p_{-},\;p_{0},\;p_{+},\;\bar{s}$, and $\bar{s}\pm 1.96\text{SEM}$ (the 95% confidence interval). Three examples that illustrate how the sentiment properties are mapped into the graphical features are shown in Fig 9. The top sentiment bar corresponds to the ‘thumbs down sign’ emoji, and indicates negative sentiment, with high confidence. The middle bar represents the estimated sentiment of the ‘flushed face’ emoji. The sentiment is neutral, close to zero, where both negative and positive sentiment are balanced. The bottom bar corresponds to the ‘chocolate bar’ emoji. Its sentiment score is positive, but its standard error bar extends into the neutral zone, so we can conclude with high confidence only that its sentiment is not negative.

### Welch’s t-test

Welch’s t-test [31] is used to test the hypothesis that two populations have equal means. It is an adaptation of Student’s t-test, but is more reliable when the two samples have unequal variances and sample sizes. Welch’s t-test is also robust for skewed distributions and even more for large sample sizes [32].

Welch’s t-test defines the t statistic as follows:

$$\begin{array}{c} t=\frac{{\bar{x}}_{1}-{\bar{x}}_{2}}{\sqrt{\frac{{\text{SD}}_{1}^{2}}{N_{1}}+\frac{{\text{SD}}_{2}^{2}}{N_{2}}}}\;. \end{array}$$

The degrees of freedom, *ν*, are estimated as follows:

$$\begin{array}{c} \nu \approx \left\lfloor \frac{{\left(\frac{{\text{SD}}_{1}^{2}}{N_{1}}+\frac{{\text{SD}}_{2}^{2}}{N_{2}}\right)}^{2}}{\frac{{\text{SD}}_{1}^{4}}{N_{1}^{2}\left(N_{1}-1\right)}+\frac{{\text{SD}}_{2}^{4}}{N_{2}^{2}\left(N_{2}-1\right)}}\right\rfloor \;, \end{array}$$

where ⌊⌋ denotes the approximate degrees of freedom, rounded down to the nearest integer. Once the t value and the degrees of freedom are determined, a p-value can be found from a table of values for Student’s t-distribution. For large degrees of freedom, *ν* > 100, the t-distribution is very close to the normal distribution. If the p-value is below the threshold of statistical significance, then the null hypothesis is rejected.

### Pearson and Spearman correlations

We need to correlate two properties of the Emoji Sentiment Ranking with other data. In the first case we correlate the emojis ranked by occurrence to the Emojitracker list—the property of the list elements is the number of occurrences. In the second case we correlate the emojis ranked by sentiment to subsets of emojis from the 13 different languages—the property of the list elements is the sentiment score.

For any two lists *x* and *y*, of length *n*, we first compute the Pearson correlation coefficient [29]:

$$\begin{array}{c} r\left(x,y\right)=\frac{\sum_{i=1}^{n}\left(x_{i}-\bar{x}\right)\left(y_{i}-\bar{y}\right)}{\sqrt{\sum_{i=1}^{n}{\left(x_{i}-\bar{x}\right)}^{2}\sum_{i=1}^{n}{\left(y_{i}-\bar{y}\right)}^{2}}}\;, \end{array}$$

where $\bar{x}$ and $\bar{y}$ are the list’s mean values, respectively. The Spearman’s rank correlation coefficient [30] is computed in the same way, the property values of the *x* and *y* elements are just replaced with their ranks. In both cases we report the correlation coefficients at the 1% significance level.

### Agreement measures

In general, an agreement can be estimated between any two methods for generating data. In our case we want to estimate the agreement between humans when annotating the same tweets for sentiment. A comparison of agreements between different datasets gives some clue about how difficult the task is. There are different measures of agreement, and to get a robust estimate of the differences, we apply three well-known measures.

Krippendorff’s *Alpha*-reliability [33] is a generalization of several specialized agreement measures. It works for any number of annotators, is applicable to different variable types and metrics (e.g., nominal, ordered, interval, etc.), and can handle small sample sizes. *Alpha* is defined as follows:

$$\begin{array}{c} \text{Alpha}=1-\frac{D_{o}}{D_{e}}\;, \end{array}$$

where *D*_*o*_ is the observed disagreement between the annotators, and *D*_*e*_ is the disagreement expected by chance. When the annotators agree perfectly, *Alpha* = 1, and when the level of agreement equals the agreement by chance, *Alpha* = 0. The two disagreement measures are defined as follows:

$$\begin{array}{c} D_{o}=\frac{1}{N}\sum_{c,c^{\prime }}N\left(c,c^{\prime }\right)\cdot \delta^{2}\left(c,c^{\prime }\right)\;, \end{array}$$

$$\begin{array}{c} D_{e}=\frac{1}{N(N-1)}\sum_{c,c^{\prime }}N\left(c\right)\cdot N\left(c^{\prime }\right)\cdot \delta^{2}\left(c,c^{\prime }\right)\;. \end{array}$$

The arguments, *N*,*N*(*c*, *c*′),*N*(*c*), and *N*(*c*′), refer to the frequencies in a coincidence matrix, defined below. *δ*(*c*, *c*′) is a difference function between the values of *c* and *c*′, and depends on the metric properties of the variable. In our case, for the discrete sentiment variables *c* and *c*′, the difference function *δ* is defined as:

$$\begin{array}{c} \delta \left(c,c^{\prime }\right)=\left|c-c^{\prime }\right|\;\;\;\;c,c^{\prime }\in \left\{-1,0,+1\right\}\;. \end{array}$$

In [33], this is called the *interval* difference function. Note that the function attributes a disagreement of 1 between the *negative* (or *positive*) and the *neutral* sentiment, and a disagreement of 2 between the *negative* and *positive* sentiments.

A **coincidence matrix** tabulates all the pairable values of *c* from two different annotators into a *k*-by-*k* square matrix, where *k* = |*c*|. Unlike a contingency matrix (used in association and correlation statistics) which tabulates pairs of values, a coincidence matrix tabulates all the pairable values. A coincidence matrix omits references to annotators. It is symmetrical around the diagonal, which contains all the perfect matches. A coincidence matrix has the following general form:

$$\begin{array}{c} \begin{array}{ccccc} & & c^{\prime } & & \sum \\ & . & . & . & \\ c & . & N(c,c^{\prime }) & . & N(c)\\ & . & . & . & \\ \sum & & N(c^{\prime }) & & N \end{array} \end{array}$$

Here *c* and *c*′ range over all possible values of the variable. In a coincidence matrix, each labeled unit is entered twice, once as a (*c*, *c*′) pair, and once as a (*c*′, *c*) pair. *N*(*c*, *c*′) is the number of units labeled by the values *c* and *c*′ by different annotators, *N*(*c*) and *N*(*c*′) are the totals for each value, and *N* is the grand total. Note that *N* is two times the number of units labeled by the different annotators.

In the case of sentiment annotations, we have a 3-by-3 coincidence matrix. Two example matrices are shown in Tables 9 and 10. Note that both coincidence matrices in Tables 9 and 10 are symmetric around the diagonal, and that the totals *N* are two times larger than in Table 3 because each annotated tweet is counted twice.

**Table 9:** Coincidence matrix for tweets with emojis.

Sentiment	*Negative*	*Neutral*	*Positive*	Total
*Negative*	1,070	354	196	1,620
*Neutral*	354	902	725	1,981
*Positive*	196	725	2,572	3,493
Total	1,620	1,981	3,493	7,094

**Table 10:** Coincidence matrix for tweets without emojis.

Sentiment	*Negative*	*Neutral*	*Positive*	Total
*Negative*	15,356	7,777	3,004	26,137
*Neutral*	7,777	23,670	10,921	42,368
*Positive*	3,004	10,921	21,624	35,549
Total	26,137	42,368	35,549	104,054

In machine learning, a classification model is automatically constructed from the training data and evaluated on a disjoint test data. A common, and the simplest, measure of the performance of the model is *Accuracy*, which measures the agreement between the model and the test data. Here, we use the same measure to estimate the agreement between the pairs of annotators. *Accuracy* is defined in terms of the observed disagreement *D*_*o*_:

$$\begin{array}{c} \text{Accuracy}=1-D_{o}=\frac{1}{N}\sum_{c}N\left(c,c\right)\;. \end{array}$$

*Accuracy* is simply the fraction of the diagonal elements of the coincidence matrix. Note that it does not account for the (dis)agreement by chance, nor for the ordering between the sentiment values.

Another, more sophisticated measure of performance, specifically designed for 3-class sentiment classifiers [12], is $\bar{F_{1}}$(−, +):

$$\begin{array}{c} \bar{F_{1}}\left(-,+\right)=\frac{F_{1}\left(-\right)+F_{1}\left(+\right)}{2}\;. \end{array}$$

$\bar{F_{1}}$(−, +) implicitly takes into account the ordering of the sentiment values by considering only the *negative* (−) and *positive* (+) labels, and ignoring the middle, *neutral* label. In general, *F*_1_(*c*) (known as the F-score) is a harmonic mean of precision and recall for class *c*. In the case of a coincidence matrix, which is symmetric, the ‘precision’ and ‘recall’ are equal, and thus *F*_1_(*c*) degenerates into:

$$\begin{array}{c} F_{1}\left(c\right)=\frac{N(c,c)}{N(c)}\;. \end{array}$$

In terms of the annotator agreement, *F*_1_(*c*) is the fraction of equally labeled tweets out of all the tweets with label *c*.
